# The effect of 16S rRNA region choice on bacterial community metabarcoding results

**DOI:** 10.1038/sdata.2019.7

**Published:** 2019-02-05

**Authors:** Yu. S. Bukin, Yu. P. Galachyants, I. V. Morozov, S. V. Bukin, A. S. Zakharenko, T. I. Zemskaya

**Affiliations:** 1 Limnological institute SB RAS, 3, 664033 Irkutsk, Russia; 2 Irkutsk Scientific Center SB RAS, 664033 Irkutsk, Russia; 3 Institute of Chemical Biology and Fundamental Medicine SB RAS, 630090, Novosibirsk, Russia

**Keywords:** Water microbiology, Metagenomics

## Abstract

In this work, we compare the resolution of V2-V3 and V3-V4 16S rRNA regions for the purposes of estimating microbial community diversity using paired-end Illumina MiSeq reads, and show that the fragment, including V2 and V3 regions, has higher resolution for lower-rank taxa (genera and species). It allows for a more precise distance-based clustering of reads into species-level OTUs. Statistically convergent estimates of the diversity of major species (defined as those that together are covered by 95% of reads) can be achieved at the sample sizes of 10000 to 15000 reads. The relative error of the Shannon index estimate for this condition is lower than 4%.

## Background & Summary

Modern microbiome studies often rely on the analysis of 16S ribosomal RNA sequences for the taxonomic identification of bacterial and archaeal strains^1^. This kind of analysis has become a *de facto* standard for prokaryotic taxonomy. The 16S rRNA gene is approximately 1600 base pairs long and includes nine hypervariable regions of varying conservation (V1-V9)^1–3^. More conservative regions are useful for determining the higher-ranking taxa, whereas more quickly evolving ones can help identify genus or species.

Metabarcoding using 16S rRNA marker is widespread in the studies of various microbial communities^4–6^.The introduction of the next-generation sequencing techniques^7–9^ has led to novel applications of metabarcoding methods. In particular, increased read counts have allowed for quantitative estimates of the microbial community composition. Another advantage of NGS-based metabarcoding is that quantitative analysis has become available for communities of uncultured microbes. Yet, using NGS technologies has its limitations, caused chiefly by shorter read length. Its most important impact is the decreased precision of species identification.

Earlier metabarcoding works were performed using 454 Life Sciences sequencer^10^ which produces reads up to 800 bp long, but this platform was discontinued by Roche in 2015. Most current work is based on the Illumina platform^11^, which produces single-end reads only up to 350 bp and paired-end reads up to 2 × 300–350 bp. As NGS reads are about one and a half times shorter than Sanger ones at best, they require a much more rigorous choice of the 16S rRNA region to precisely and comprehensively describe the diversity of a bacterial community. Clustering of a short conservative region has insufficient resolution to detect the fine differences between strains that occupy slightly different niches. In other words, separating the sample into too many very small OTUs does not decrease analysis quality, but separating it into OTUs that are too crude does precisely that by lumping several species with potentially different ecology into a single OTU. Therefore, using a more variable region can detect finer taxonomic differences between communities, which in turn can be used to describe finer differences in their functioning. It should be also be mentioned that increasing read length for Illumina and 454 Life Sciences sequencers leads to decreasing the quality, *i.e.*, accuracy, of read sequences. Based on all these factors, researchers must design their taxonomic experiments using correct region and sequencing coverage so that it could a produce statistically sound description of both the bacterial species present in the sample and their relative numbers.

A series of experimental works on artificial microbial communities using NGS metabarcoding methods^12–14^ has shown that a choice of 16S rRNA region can significantly affect the estimates of taxonomic diversity. In particular, using different regions leads to estimated proportions of taxa different from each other and from the known true composition.

One of the possible approaches to solving this problem is to use the experience of studying the same communities using different 16S rRNA regions. The microbiome of lake Baikal is well-studied by microbiologists. Specifically, metabarcoding works have been performed for the communities of the water column^15–17^, bottom sediments^18–22^, and communities associated with certain baikalian organisms^23,24^. These works have shown that the bacterial biodiversity in the lake is extremely high. High biodiversity in Lake Baikal can be explained by the presence of multiple distinct niches varying in terms of environmental conditions.

Our main aim was a comparative analysis of the bacterial communities from distinct ecotopes in Lake Baikal as revealed by metabarcoding analysis using V2-V3 and V3-V4 fragments of the 16S rRNA gene. The total DNA was extracted from biological samples, amplified independently using primer pairs designed for V2-V3 and V3-V4 fragments, and sequenced, after which community composition and bacterial diversity were estimated using bioinformatics methods. We assumed that the region that produces the highest diversity will be most useful for further metabarcoding studies. Analysis was performed on the communities inhabiting contrasting biotopes in Lake Baikal. The bottom sediment is an organically rich substrate, whereas the water column is relatively poor in this regard.

## Methods

### Sampling

In this work, we studied the bacterial communities of Lake Baikal’s water column and bottom sediment. Water column samples were taken in July 2013 near «Gorevoy Utes» underwater oil seep (central basin, 53.3045170°N 108.3919330°E, depth 855 m) – zone I (R-6) and near the «Bolshoy» mud volcano (southern basin, 51.877900°N 105.550517°E, depth 1370 m) – zone II (R-9). The samples of the bottom sediments associated with surface methane hydrate sediments were taken in July 2015 at the «Akademicheskiy khrebet» station (central basin, 53.399782°N 107.891370°E, depth 536 m). All the samples were taken from aboard the «Akademik Vereschagin» research vessel using SBE 32 Carousel Water Sampler bathometer system (USA) and gravity corer. Full description and sample identifiers are found in Table 1.

### DNA extraction

Eight samples from zone I (taken at depths of 0, 50, 100, 200, 300, 500, 700, and 855 m) and five samples from zome II (taken at depths of 0, 50, 100, 700, and 1370 m) were used for DNA extraction. Five liters of water from each sample were filtered using nitrocellulose filters (25 mm diameter, 0.2 micron pores, «Millipore», Germany) using a squeeze pump. Filters were placed in TE buffer (10 mM Tris-HCl, pH 7.4; 1 mM EDTA, pH 8.0), frozen at −20 °C and transported to the laboratory.

Sediment sample (100 g) was aseptically taken aboard the vessel from the 150–185 cm core layer, homogenized, frozen in liquid nitrogen at −196 °C and transported to the laboratory.

DNA extraction from all the samples was performed by lysozyme treatment. Sediment samples were homogenizedin an agate mortar with SiC prior lysis. For DNA extraction, phenol-chlorophorm technique^25^ was used with several modifications^26^. Four independent DNA extractions were carried out for each sample. In addition, a negative control (DNA extraction with sterile TE-buffer) was performed for each independent DNA extractions to ensure that no contamination with exogenous amplifiable DNA occurred during the different stages of sample treatment. Concentration and quality of extracted DNA were measured with a spectrophotometer SmartSpec Plus (BioRad, USA). DNA was stored at −70 °C until further analysis.

### Amplification and sequencing

B_V23 and Pro_V34 bacterial 16 rRNA gene fragments were amplified using universal primers (Table 2). Phusion Hot Start II High-Fidelity DNA polymerase (Thermo Scientific #F-549S) with High-Fidelity Buffer was used for the amplification. After optimizing the polymerase chain reaction (PCR) conditions (thermal profile and Mg^2+^ concentration), the required minimum of PCR cycles was adjusted for every DNA sample, thereby preventing the plateau effect in concentrations of the products. For this purpose, concentration of the PCR products was controlled via capillary electrophoresis on a Shimadzu Multi-NA instrument (DNA-12000 reagent kit). Libraries for Illumina MiSeq analysis were prepared with NEBNext Ultra II DNA Library Prep Kit (New England Biolabs). The libraries were analysed using the Illumina MiSeq Standard Kit v.3 (Illumina) at the Genomics Core Facility, Institute of Chemical Biology and Fundamental Medicine, Siberian Branch of the Russian Academy of Sciences (Novosibirsk). All data 16S rRNA fragments were deposited in the NCBI Sequence Read Archive, bottom sediment (Data Citation 1) and water (Data Citation 2).

### Read analysis

Read analysis was conducted in Mothur v.1.34.4 software according to MiSeq SOP recommendations^27^. R1 and R2 sequences corresponding to ribosomal RNA amplicons were merged into contigs with the mothur merge.contigs command, and resulting fragments were filtered by quality in non-overlapping regions as having no more than five sites with a Phred-value <= 15. Scripts used to filter MiSeq data in a quality manner are available at: https://github.com/yuragal/mothur-scripts. Filtered sequences were aligned, clustered, and identified taxonomically using the SILVA 123 databases (http://arb-silva.de). Reads were clustered into OTU at genetic distances of 0.03, which is a typical interspecies distance within a genus. The information on the species-rank OTU abundance (number of reads per OTU) was collated in a single table.

### Statistic comparisons of species diversity

Statistical convergence of species diversity estimates was measured using the bootstrap index^28^ which shows the potential amount and proportion of undetected species in the community (underestimated α-diversity).

Species-rank OTU abundance was used to calculate Shannon^29^ and Simpson indices^29^ of community biodiversity. Their confidence intervals and relative errors were calculated using the bootstrap algorithm proposed in^30^. Correlations between sample sizes, values of indices, and relative errors were measured by non-parametric Spearman coefficients^31^ and correlation significance was tested using Spearman statistics.

Potential number of species in communities (hidden α-diversity - hidden species richness) was evaluated using Chao1^32^ and ACE^33^ indices. Standard errors for these indices were determined by methods^32,33^.

The significance of differences between average value of Shannon, Simpson, Chao1 and ACE indices identified by V2-V3 and V3-V4 fragments was estimated using paired modification of the Wilkinson-Mann-Whitney nonparametric criterion^34^.

Qualitative and quantitative comparison of the community composition at different levels of taxonomic organization (phylum, class, order, family) were carried out using Nonmetric Multidimensional Scaling - NMDS^35^ with Bray-Curtis distance metric^36^, Gower distance metric^37^ and Jaccard distance metric^36^. Before analysis, all data were normalized by the average number of reads per sample. For analysis, V2-V3 and V3-V4 OTUs were combined into one dataset (taxonomy association). The degree of differences in the taxonomic composition of communities by the factor V2-V3 and V3-V4 fragments for Bray-Curtis, Gower and Jaccard distance metric was estimated using the R2 - squared covariation coefficient (R2 = 1-ss_w/ss_t, where ss_w and ss_t are within-group and total sums of squares). R2 reliability was estimated using permutation test (1000 permutation)^38^. Estimates of taxon representation in samples (number of reads per taxon) were visualized in the form of heat maps, where the rows and columns were clustered using the «average» method based on the distance matrix calculated with Bray-Curtis, Gower or Jaccard distance metrics.

### Statistic comparisons of the genetic diversity estimates

The proportion of mismatched nucleotides between sequences (p-distance) was used as a genetic distance metric. For each OTU, the sequences that have a minimal sum of distances to others within the same OTU were selected as representative. The pairwise p-distance distributions and nucleotide diversity (average p-distance) estimates^39^ were built for V2-V3 and V3-V4 representative sequence samples. The significance of differences between average p-distances was estimated using of the Wilkinson-Mann-Whitney nonparametric criterion^34^.

The closest full-length 16S rRNA sequences for each representative sequence from both regions were found in the SILVA 123 database using mothur software. In this way, we have created two samples of full-length rRNAs describing OTUs detected in the analysis of V2-V3 and V3-V4 fragments. These two samples were pooled into a single dataset for which we then built the p-distance matrix and performed NMDS^35^ to detect the similarity between OTU and species sets detected in the independent analyses of V2-V3 and V3-V4 fragments.

Statistical analyses were performed using the «ape»^40^, «pegas»^41^, «gplots» and «vegan»^42^ R packages.

### Code availability

Scripts used to filter MiSeq data in a quality manner are available at: https://github.com/yuragal/mothur-scripts.

Scripts for R programming language used for statistic comparisons of species diversity and statistic comparisons of the genetic diversity are available at: https://github.com/barnsys/16S_rRNA_bacterial_communities_analysis. These scripts were created on the basis of tutorials to the vegan package^43,44^.

## Data Records

All data on V2-V3 and V3-V4 fragment 16S rRNA were deposited in the NCBI Sequence Read Archive, bottom sediment (Data Citation 1) and water (Data Citation 2).

## Technical Validation

The filtered dataset for the V2-V3 16S rRNA fragment included 118232 reads with the average length of 205 base pairs. It was clustered into 2716 species-level OTUs, 1070 of which included more than one read. Singleton OTUs included 1.4% of the reads. The V3-V4 dataset included 22191 reads with an average length of 443 base pairs clustered into 1615 species-level OTUs, 509 of them including more than one read. Singleton OTUs included 5.2% of the reads. These numbers suggest an acceptable quality of sequencing and initial data filtering stages. Singleton OTUs were excluded from further analyses.

For analyzing the convergence of α-diversity estimates, the OTUs were sorted from most to least abundant (*i.e.,* in the order of decreasing read counts). Analysis of the α-diversity convergence using the bootstrap index has shown that the proportion of the underestimated OTUs (that is, those that could be present in the biological sample, but went undetected because of incomplete sequencing) among top-ranking OTUs, including 95% of reads, does not exceed 16%, which is under the 20% level considered acceptable for biological studies. Further analyses were performed on this pool of top OTUs covering 95% of reads. It included 251 OTUs for the V2-V3 16S rRNA region and 171 OTUs for the V3-V4 region.

**Figure 1:**
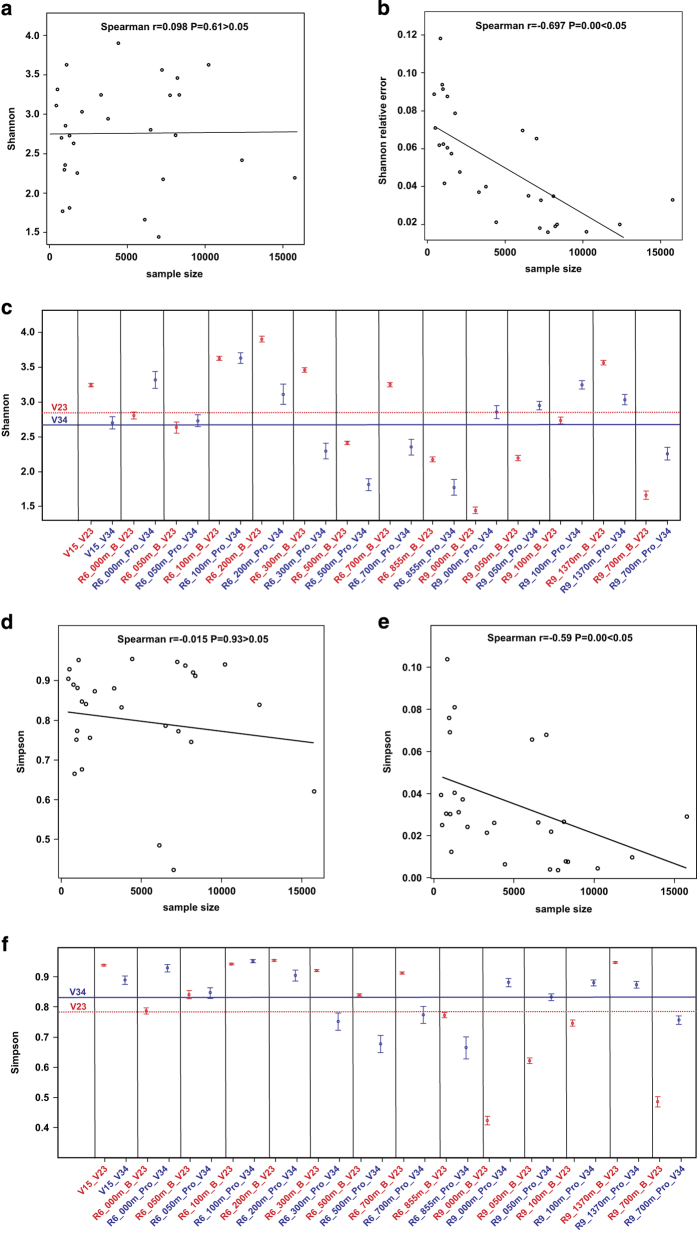
Comparative analysis of indices Shannon and Simpson. (**a**) Dependence of the Shannon index from the sample size for V2-V3 and V3-V4 16S rRNA fragments. (**b**) Dependence of the Shannon relative error from the sample size for V2-V3 and V3-V4 16S rRNA fragments. (**c**) The per-sample Shannon indices, point bars indicate 95% CI, red line – average value for V2-V3 fragments, blue line – average value for V3-V4 fragments. (**d**) Dependence of the Simpson index from the sample size for V2-V3 and V3-V4 16S rRNA fragments. (**e**) Dependence of the Simpson relative error from the sample size for V2-V3 and V3-V4 16S rRNA fragments. (**f**) The per-sample Simpson indices, point bars indicate 95% CI, red line – average value for V2-V3 fragments, blue line – average value for V3-V4 fragments. V2-V3 fragments 16S rRNA – red pointer, V3-V4 fragments – blue pointer. The encoding of the samples is indicated in Table 1.

The values for Shannon biodiversity indices in all samples with both regions ranged from 1.5 to 4 (Fig. 1). In eight out of 14 samples, these indices were higher when estimated using the V2-V3 fragment, while in six out of 14 samples, the V3-V4 fragment produced higher values. Shannon indices averaged 2.79 for the V2-V3 region and 2.72 for the V3-V4 region. Testing with the Wilkinson-Mann-Whitney index showed (Table 3) that the averages of the Shannon indices did not differ significantly between the regions. Thus, metabarcoding with either V2-V3 or V3-V4 16S rRNA fragments yield similar species diversity estimates.

Comparison of Shannon indices from different samples (Fig. 1) demonstrated that the bacterial diversity in water columns vary with depth. At some depths, the values were close to the minimum (1.5), whereas at others, bacterial communities were highly diverse (4.0). Despite the variety in primary carbon sources, Shannon indices in bottom sediment communities were close to the average at 3.24 and 2.7 for the V2-V3 and V3-V4 regions, respectively.

Correlation analysis (r = 0.098, P-value = 0.61 > 0.05) shows that there is no correlation between Shannon indices and read counts. It means that read counts are sufficient for characterizing community diversity in the studied samples in up to 95% of the major OTUs. Were the coverage of the community insufficient, Shannon indices would increase with the read count based on sampling more and rarer species, and thus there would be a significant positive (r > 0) correlation between these two values.

Relative error of the Shannon index values, estimated for 95% confidence intervals, did not exceed 12%, which is, again, under the acceptable level of 20% (Fig. 1). Correlation between the Shannon index relative error and read count is significant and negative (r = −0.697, P-value = 0.00 <0.05). This means that increasing read counts leads to increasing precision of the Shannon index estimate. Using samples of at least 5000 reads, it did not exceed 8%, while increasing the sample size to 10000 reads further decreased it to less than 4%, which is a positive result for complex natural samples. It is important to note that the convergent estimates of species diversity in species-rich communities usually require higher read counts. In our work, a number of the samples with Shannon index values of more than 3.5 (which is practically as high as possible for a biological sample) had relative errors of roughly 3% (Fig. 1). These communities were characterized using datasets of 10000–15000 reads. Thus, we can conclude that these read counts are sufficient for the metabarcoding analysis of the 95% top OTUs of a bacterial community, and higher sample sizes are unnecessary.

In all samples, the Simpson biodiversity indices ranged from 0.42 to 0.94 for both V2-V3 and V3-V4 regions (Fig. 1). For eight out of 14 samples, these indices were higher for V3-V4 fragment, while in six out of 14 samples, the V2-V3 region produced higher values (mean value 0.79 for the V2-V3 region and 0.82 for the V3-V4 region). Shannon biodiversity indices reveal a different pattern compared to Simpson index. However the testing with the paired Wilkinson-Mann-Whitney test demonstrates (Table 3) that the averages of the Simpson indices did not differ significantly from Shannon ones. Thus, metabarcoding with either V2-V3 or V3-V4 16S rRNA fragments yields roughly the same species diversity estimates both with Simpson and Shannon indices. The dependence of the Simpson index values on the samples is similar to the patterns observed for the Shannon index (Fig. 1).

Correlation analysis (r = 0.015, p = 0.93 > 0.05) shows no correlation between Simpson indices and read counts. The same is observed for the Shannon indices and this is explained by the similar reasons. Relative error of the Simpson index values, estimated for 95% confidence intervals did not exceed 11% (Fig. 1). Correlation between the Simpson index relative error and read count is significant and negative (r = −0.59, p ~ 10^5^ < 0.05). This means that increase of read counts results in increase of precision of the Simpson index estimate. The relationship between relative errors of the Simpson indices and the number of reads per sample can be explained by similar reasons as for the Shannon indices.

**Figure 2:**
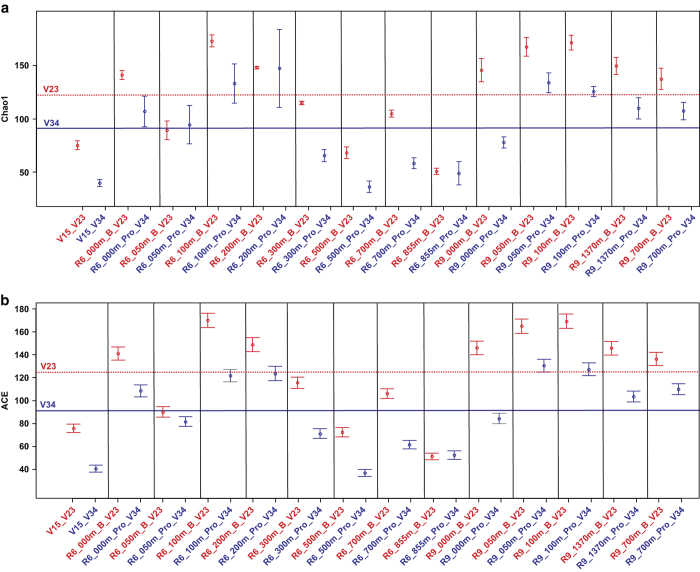
Comparative analysis of indices Chao1 and ACE. (**a**) The per-sample Chao1 indices, the diagram features show standard errors, red line – average value for V2-V3 fragments blue line – average value for V3-V4 fragments. (**b**) The per-sample ACE indices, the diagram features show standard errors, red line – average value for V2-V3 fragments, blue line – average value for V3-V4 fragments. V2-V3 fragments 16S rRNA – red pointer, V3-V4 fragments 16S rRNA – blue pointer. The encoding of the samples is indicated in Table 1.

An analysis of the Chao1 and ACE indices (Fig. 2) shows that in most cases the hidden species richness computed by V2-V3 region is higher than that computed by V3-V4 region. For the V2-V3 region, the Chao1 index values varied from 51 to 172 (mean value 124), the ACE index values varied from 48 to 163 (mean value 123). For the V3-V4 region, the Chao1 index values varied from 36 to 147 (mean value 91), the ACE index values varied from 36 to 130 (mean value 89). Wilkinson-Mann-Whitney test shows the average value of both Chao1 and ACE indices for V2-V3 region are higher than those for V3-V4 region (Table 3). Statistically confirmed that V2-V3 region for metabarcoding studies of microbial communities gives greater resolution at low clustering thresholds (the species level, 0.03 in our case) than V3-V4 region.

**Figure 3:**
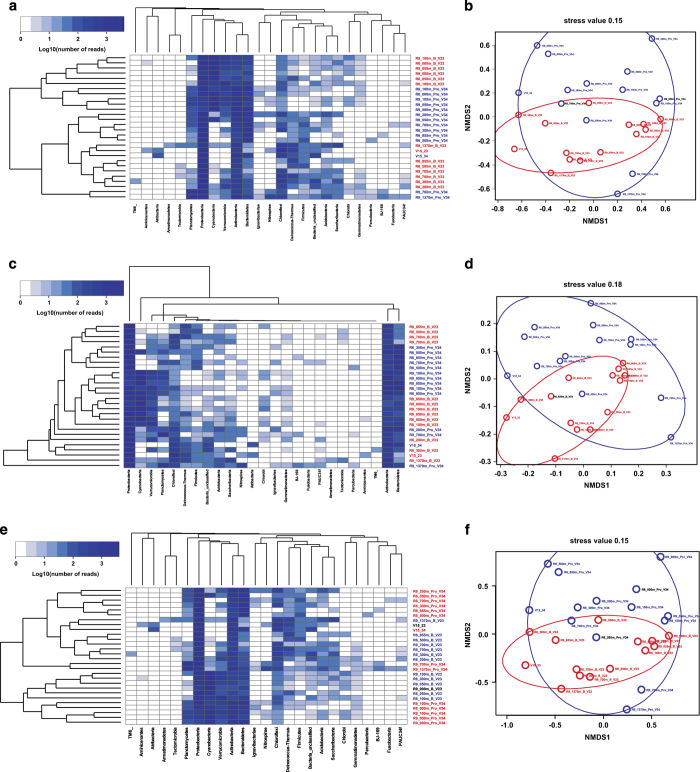
Comparative analysis of microbial communities, performed at the phylum level. (**a**) Heat map for phylums representation and clustering samples with the Bray-Curtis distance metric. (**b**) NMDS scatter plot clustering of samples on Bray-Curtis distance metric. (**c**) Heat map for phylums representation and clustering samples with the Gower distance metric. (**b**) NMDS scatter plot clustering of samples on Gower distance metric. (**e**) Heat map for phylums representation and clustering samples with the Jaccard distance metric. (**f**) NMDS scatter plot clustering of samples on Jaccard distance metric. V2-V3 fragments – red pointer, V3-V4 fragments – blue pointer. The encoding of the samples is indicated in Table 1.

Moving to analysis of phylotypes, we were interested in correlation of OTUs obtained using V2-V3 and V3-V4 fragments mapped to bacterial taxons of higher level, such as phylum, class, order and family. Analysis of Bray-Curtis distances (which is semi metric distances index) at the phylum level (Fig. 3) shows that clouds of points overlaps on the NMDS scatterplot. There is also no clear clustering of samples on the heat map dendrogram. The covariance coefficient R2 = 0.12 (Table 4) shows weak differences in the presence or absence of common phyla while comparing samples either by V2-V3 or V3-V4 regions, although these differences were significant (p = 0.04 < 0.05). The Gower distance (which is quantitative distances index) shows a similar results at the level of phyla (R2 = 0.14, p = 0.04 < 0.016) (Fig. 3 and Table 4). Consequently, number of reads per phyla in the paired comparison of samples by V2-V3 or V3-V4 regions did not differ much. With lower taxonomic ranks (from class to family), differences between samples analyzed by V2-V3 or V3-V4 regions become larger (Table 4). Jaccard metric of distance (which is metric distances index) showed a result very similar to the analysis based on the Bray Curtis index (Fig. 3 and Table 4). The close results of the Bray Curtis and Jaccard indices are related to the fact that data were normalized by the average number of reads per sample At lower taxonomic ranks, increase differences both in terms of the qualitative metric and the quantitative metric of distance.

**Figure 4:**
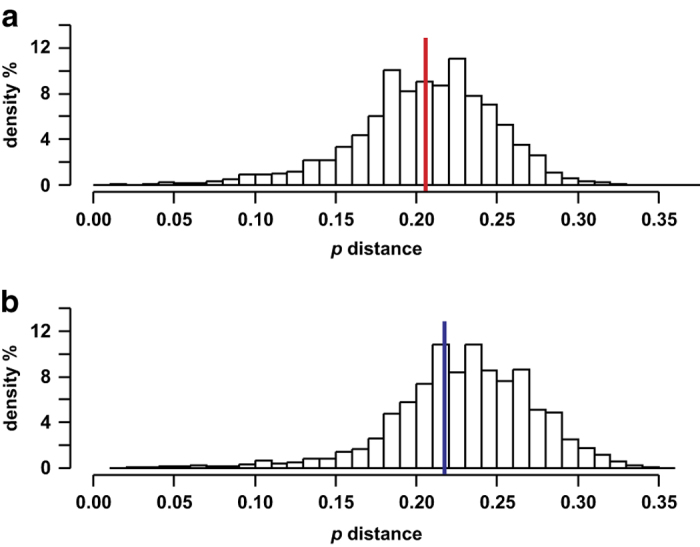
The histograms of pairwise genetic distances (p-distances) between the representative sequences of a 95% major OTU pool. (**a**) V2-V3 fragments 16S rRNA, red line - average distance. (**b**) V3-V4 fragments 16S rRNA, blue line - average distance.

Representative sequence sets had different genetic diversities for the OTUs generated using V2-V3 and V3-V4 fragments (Fig. 4). Analysis of the V2-V3 region has produced lower average genetic distances than the V3-V4 fragment did (0.204 vs 0.228). According to the Wilkinson-Mann-Whitney test, the differences between genetic distance samples are significant (P-value = 0.00 < 0.05). A histogram of the pairwise distances for the V3-V4 fragment is skewed to the right compared to that for the V2-V3 fragment. The latter also exhibited a quicker increase in the genetic distance frequencies in the area of lower values. Thus, the fragment of the 16S rRNA gene that includes V2 and V3 regions accumulates mutations quicker than V3 and V4 regions do during early stages of bacterial speciation. In this work, species-level OTUs were detected at the 0.03 genetic distance threshold, which is in the lower portion of the histogram. Therefore, V2-V3 16S rRNA fragments are better suited for distinguishing closely related species (for example, species within a genus). In other words, OTUs will be smaller when using V2-V3 fragments than when using V3-V4 fragments. This is, in fact, the reason why there are 271 and 171 OTUs, respectively, although analyses of both fragments produce convergent Shannon diversity index estimates. Analyzing the V2-V3 region has led to detection of 32% more species in the top 95% of the OTU reads pool than V3-V4 fragments. Obviously, using V3-V4 fragments does not allow for distinguishing some species-level OTUs, erroneously merging them into a single species. We can therefore conclude that V2-V3 16S rRNA fragments are more appropriate for the metabarcoding works aiming at detecting species in the bacterial community.

**Figure 5:**
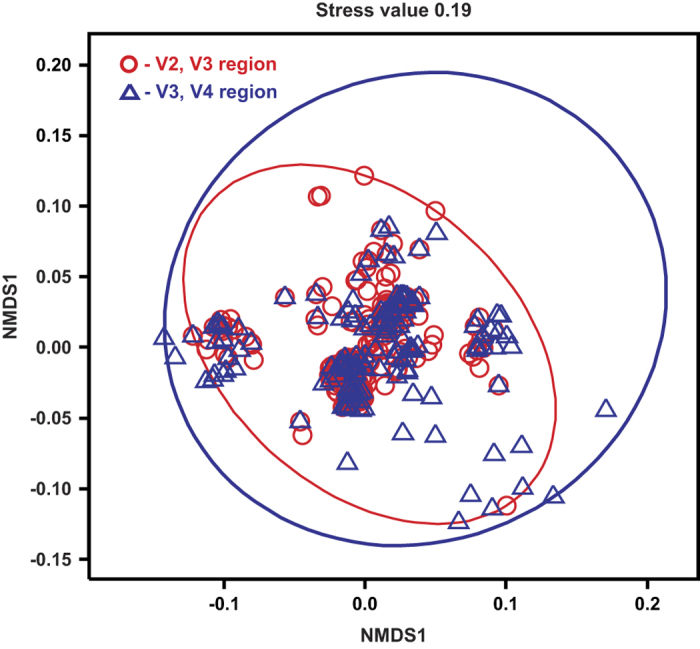
Scatter plot based on the matrix of the distances between full-length 16S rRNA sequences from the SILVA database closest to the representative sequences of OTUs found in V2-V3 and V3-V4 analyses. The blue ellipse includes all OTUs detected using V3-V4 fragment analysis, while a red one includes all V2-V3 OTUs.

Comparison of species-level OTUs detected with V2-V3 and V3-V4 16S rRNA was performed using the corresponding full-length 16S rRNA sequences from the SILVA database (Fig. 5). It can be seen that OTUs detected with both fragments form several dense clusters of closely related OTUs. In a number of cases, the points corresponding to similar OTUs detected with different 16S rRNA fragments overlap precisely, in which case species are exactly matched. In other, quite numerous, cases they do not overlap; often, there are no V3-V4-based OTUs close to those detected with V2-V3 fragments. This means that taxa detected using the V2-V3 region are genetically different from those detected with the V3-V4 fragment owing to differences in their genetic variance described previously. V3-V4 fragment analysis has merged many of the species (OTUs) separated in V2-V3 clustering into a single OTU. The results of the multidimensional scaling of the full-length 16S rRNAs’ genetic distances match the results of the analysis of the genetic variance of the V2-V3 and V3-V4 fragments.

## Usage Notes

Our results show that targeting V2-V3 or V3-V4 16S rRNA fragments result in similar estimates of community diversity in metabarcoding studies as measured by the Shannon and Simpson indices. From the first view, this means there is little advantage of one index over another in practice. Yet, Shannon and Simpson indices themselves or their comparisons do not tell much about how similar or different the communities are in their species composition^45^. Any diversity index used in this work, including Shannon and Simpson ones, depends on the number of species and the uniformity of their abundance or biomass. The more species there are in the community and the more evenly their abundance is distributed, the higher Shannon and Simpson indices will be. Differences in the species spectrum (the number of shared and non-shared species) do not affect it. On the other hand, the analysis of the indices of the expected species richness such as Chao1 and ACE showed that the number of species identified by V2-V3 fragments is larger than V3-V4 fragments. When the species clusters are determined at the level of genetic distances of 3%, V2-V3 fragments has a higher resolution than fragment V3-V4 fragments.

A different question is much more important for comparative ecology: are the species similar between samples or are they different? One of the most commonly used measures of community similarity, Bray-Curtis dissimilarity, does depend on the counts of shared and non-shared specimens in two communities. Therefore, in metabarcoding studies, imprecise estimates of the biodiversity with any index (for example, Shannon) are less important than possible artifacts of processing raw data into the lists of species. Considering that a given bacterial species can carry out specific functions in the community by existing in specific niches, measures of community similarity or differences, such as Bray-Curtis dissimilarity, capture the differences in communities’ ecology and biochemical capabilities. Detection of species (strictly speaking, species-level OTUs) in metabarcoding is based on genetic distances and clustering methods. If a marker, for example, a 16S rRNA fragment turns out to be too conserved, the genetic distances will be too low, different species will be lumped into a single OTU, and the information regarding the differences in structure or functioning of the communities will be lost. Two or more potentially different OTUs occupying different niches (reacting variably to environmental differences between samples) do not inform the researcher about differences between communities when they are merged into a single OTU. On the other hand, splitting a single species into several different OTUs will only lead to detection of several pseudotaxa similarly reacting to the environment, which does not impact ecological conclusions. Our work shows that the V2-V3 fragment of the 16S rRNA gene is preferable for metabarcoding analyses as the V3-V4 fragment underestimates species diversity by merging several species into a single OTU.

We can consider the possibility that the difference in species spectra detected by the V2-V3 and V3-V4 16S rRNA fragments is related to primer specificity and PCR artifacts^2^. The V2-V3 primer pair is more specific for certain taxa, while the V3-V4 amplifies others’ genes better. There are two arguments against this idea: first, lesser primer specificity would lead to decreasing the abundance of particular taxa, which in turn would disrupt the distribution uniformity and decrease the Shannon index. Our results show that there is no significant difference between Shannon indices produced with either fragment, *i.e.,* that the taxa distribution uniformity is practically similar in both diversity estimates. It does not, though, exclude a rare possibility that some species’ genes were not amplified at all with V2-V3 primers, but amplified well with V3-V4 ones, or *vice versa*. Second, the two pools of major OTUs produced with two primer pairs included different species. While a number of OTUs from the V2-V3 and V3-V4 analyses matched the same full-length sequence from the SILVA database (taxonomically matching species), though some others did not. If the problem were related to primer specificity and PCR effectiveness for different taxa, practically every one of 171 OTUs detected in the V3-V4 analysis would have a counterpart among 251 OTUs from the V2-V3 run.

The reasons behind these peculiarities of the bacterial taxonomic identification performed using different 16S rRNA fragments may be related to the functions of these fragments. The functions of nine regions (V1 to V9) can be illustrated by the molecule’s three-dimensional structure^46^. 16S rRNA’s essential function is to take part in the translation process, relative to which the regions can be separated into three classes. The first of them includes V4, V5 and V6, which directly take part in the translation and are responsible for binding tRNAs and interacting with the 23 S rRNA^47–49^. A second class includes regions V3 and V7, of which the role in translation is currently understudied. A third class, V2 and V8, is responsible for maintaining the structural stability of 16S rRNA^48^. According to their functions, regions of the first group should be the most conservative, followed by more variable V3 and V7, and finally by the quickest-evolving V2 and V8. Regions of the first group will accumulate mutations slowly and, at the phylogenetic level, should be sufficiently distinct only in higher taxa, such as phyla and classes. Less conservative regions of the second group will be different between orders and families. The third class regions, V2 and V8, could distinguish genera within a family and species within a genus. In our work, one of the fragments (V2-V3) included one region from the third class and one from the second, and another (V3-V4) included regions from the second and first classes, so it was reasonable to expect that regions of V2-V3 fragment will provide a better picture of species- or genus-level resolution than will V3-V4. The results completely confirm the picture of species diversity that would be expected from the 16S rRNA regions’ conservation^50^.

The problem of lower resolution of V3-V4 fragments at the species level can be solved by reducing the threshold of genetic distances which is used for OTU clustering. Some studies^51,52^ suggest to reduce the clustering threshold to 1.3%. or 1%. In the course of the study, it would be possible to change the threshold to 2 or 1%. However, here one may meet a number of problems. The accuracy of base call in Illumina technology is significantly less than that of Sanger method. A fragment of 100 decoded nucleotides can account for 1 or 2 errors, and this exceeds the threshold level of 1 or 2%. Thus, by lowering the selection threshold by the species delimitation, one may come across the fact that new taxa will be distinguished due to sequencing errors.

One of the studies^53^ characterizing the communities of the female genital tract shows that V3-V4 fragments produced the increased α-diversity estimates such as Simpson and Chao1 indices. The conclusions of this work are based on an analysis of bacterial communities of the same biotope from different individuals (38 females). In our study, there were also several samples (Figs 1 and 2) where Simpson and Chao1 indices computed for V3-V4 data were greater than those for V2-V3 data. However, in most cases, the V2-V3 data produced α-diversity and Chao1 index values greater when compared with V3-V4 data. The communities studied in our work were sampled from contrasting biotopes of the Lake Baikal ecosystem (different depths in the water column and bottom sediment). The studied biotopes are characterized by different temperatures, concentrations of oxygen, organic matter, pH, mineralization and concentrations of biogenic elements. Therefore, the conclusions drawn from our research are likely more generally applicable than those from the work of^53^, although it is possible for some microbial communities V3-V4 fragment will better delineate the fine-grained community structure.

The bacterial communities consisted of taxa characteristic of freshwater lakes^54^ and were similar to the community composition of other Baikal areas^17,55^. In the communities, we observed a high percentage of sequences of the phyla Actinobacteria and Bacteroidetes. The presence of these bacteria in the communities may be due to their active role in the destruction of the dying diatoms, which massively develop under the ice of Lake Baikal in the spring^55,56^. In their genomes, the key enzymes and pathways for effective degradation of at least two polysaccharides, disaccharides, and amino sugars were detected^57^.

Results of this work show that for the estimation of the bacterial communities’ taxonomic diversity using 16S rRNA, the metabarcoding method with Illumina MiSeq paired-end reads technology; the V2-V3 fragment has the highest resolution for the lower-rank taxa (species and genera). Choosing this fragment for the analysis allows for more precise separation of the read pool into species-level OTUs based on genetic distances. Statistically convergent estimates of the species diversity for the major part of the community (OTUs covering 95% of reads) can be acquired using samples of 10000 to 15000 reads. In this case, relative error of the diversity index estimates will be under 4%.

## Additional information

**How to cite this article**: Bukin, Y. S. *et al*. The effect of 16S rRNA region choice on bacterial community metabarcoding results. *Sci. Data*. 6:190007 https://doi.org/10.1038/sdata.2019.7 (2019).

**Publisher’s note**: Springer Nature remains neutral with regard to jurisdictional claims in published maps and institutional affiliations.

## Supplementary Material



## Figures and Tables

**Table 1:** Information on the analyzed samples.

No	Encoding	Fragments 16S rRNA	Sampling area	Substrate type	Deeps m	*NCBI SRR*	*NCBI SRP*
1	V15_V23	23	«Akademicheskiy khrebet» station	bottom sediment	—	SRR7160311	SRP145556
2	R6_000m_B_V23	23	«Gorevoy Utes» underwater oil seep	water	0	SRR7472141	SRP102494
3	R6_050m_B_V23	23	«Gorevoy Utes» underwater oil seep	water	50	SRR7472140	SRP102494
4	R6_100m_B_V23	23	«Gorevoy Utes» underwater oil seep	water	100	SRR7472143	SRP102494
5	R6_200m_B_V23	23	«Gorevoy Utes» underwater oil seep	water	200	SRR7472142	SRP102494
6	R6_300m_B_V23	23	«Gorevoy Utes» underwater oil seep	water	300	SRR7472137	SRP102494
7	R6_500m_B_V23	23	«Gorevoy Utes» underwater oil seep	water	500	SRR7472136	SRP102494
8	R6_700m_B_V23	23	«Gorevoy Utes» underwater oil seep	water	700	SRR7472139	SRP102494
9	R6_855m_B_V23	23	«Gorevoy Utes» underwater oil seep	water	855	SRR7472138	SRP102494
10	R9_000m_B_V23	23	«Bolshoy» mud volcano	water	0	SRR7472145	SRP102494
11	R9_050m_B_V23	23	«Bolshoy» mud volcano	water	50	SRR7472144	SRP102494
12	R9_100m_B_V23	23	«Bolshoy» mud volcano	water	100	SRR7472133	SRP102494
13	R9_1370m_B_V23	23	«Bolshoy» mud volcano	water	1370	SRR7472131	SRP102494
14	R9_700m_B_V23	23	«Bolshoy» mud volcano	water	700	SRR7472132	SRP102494
15	V15_V34	34	«Akademicheskiy khrebet» station	bottom sediment	—	SRR7160312	SRP145556
16	R6_000m_B_V34	34	«Gorevoy Utes» underwater oil seep	water	0	SRR7472130	SRP102494
17	R6_050m_B_V34	34	«Gorevoy Utes» underwater oil seep	water	50	SRR7472129	SRP102494
18	R6_100m_B_V34	34	«Gorevoy Utes» underwater oil seep	water	100	SRR7472128	SRP102494
19	R6_200m_B_V34	34	«Gorevoy Utes» underwater oil seep	water	200	SRR7472127	SRP102494
20	R6_300m_B_V34	34	« Gorevoy Utes» underwater oil seep	water	300	SRR7472126	SRP102494
21	R6_500m_B_V34	34	Gorevoy Utes» underwater oil seep	water	500	SRR7472135	SRP102494
22	R6_700m_B_V34	34	«Gorevoy Utes» underwater oil seep	water	700	SRR7472134	SRP102494
23	R6_855m_B_V34	34	«Gorevoy Utes» underwater oil seep	water	855	SRR7472150	SRP102494
24	R9_000m_B_V34	34	«Bolshoy» mud volcano	water	0	SRR7472151	SRP102494
25	R9_050m_B_V34	34	«Bolshoy» mud volcano	water	50	SRR7472148	SRP102494
26	R9_100m_B_V34	34	«Bolshoy» mud volcano	water	100	SRR7472149	SRP102494
27	R9_1370m_B_V34	34	«Bolshoy» mud volcano	water	1370	SRR7472147	SRP102494
28	R9_700m_B_V34	34	«Bolshoy» mud volcano	water	700	SRR7472146	SRP102494

**Table 2:** Loci selected for the analysis and structure of oligonucleotide primers for their amplification.

Gene	Amplicon	Primer	Sequence
16S rRNA	B_V23	16S_BV2f	AGTGGCGGACGGGTGAGTAA
		16S_BV3r	AGTGGCGGACGGGTGAGTAA
	Pro_V34	MiCSQ_343FL	TATGGTAATTGTCTCCTACGGRRSGCAGCAG
		MiCSQ_806R	AGTCAGTCAGCCGGACTACNVGGGTWTCTAAT

**Table 3:** Comparison of community diversity indices.

Community diversity Indices	Mean value fot V2-V3 region	Mean value fot V3-V4 region	P_value from Wilkinson-Mann-Whitney test
Shannon index	2.79	2.72	0.62 > 0.05
Simpson index	0.79	0.82	0.76 > 0.05
***Chao1 index***	***124***	***91***	***0.0006 < 0.05***
***ACE index***	***123***	***89***	***0.0002 < 0.05***
Values in bold italics are significantly different.			

**Table 4:** Difference in community structure evaluated with V2-V3 and V3-V4 regions at different taxonomic ranks.

Taxonomic level	NMDS from Bray-Curtis distance		NMDS from Gower distance		NMDS from Jaccard distance	
	R2	P_value	R2	P_value	R2	P_value
phylum	0.12	0.04	0.14	0.016	0.11	0.036
class	0.35	0.001	0.28	0.001	0.35	0.001
order	0.35	0.001	0.38	0.001	0.35	0.001
family	0.42	0.001	0.39	0.001	0.43	0.001
