# Author Correction: The effect of 16S rRNA region choice on bacterial community metabarcoding results

**DOI:** 10.1038/s41597-022-01246-0

**Published:** 2022-03-17

**Authors:** Yu. S. Bukin, Yu. P. Galachyants, I. V. Morozov, S. V. Bukin, A. S. Zakharenko, T. I. Zemskaya

**Affiliations:** 1 Limnological institute SB RAS, 3, 664033 Irkutsk, Russia; 2 Irkutsk Scientific Center SB RAS, 664033 Irkutsk, Russia; 3 Institute of Chemical Biology and Fundamental Medicine SB RAS, 630090 Novosibirsk, Russia

Correction to: *Scientific Data* 10.1038/sdata.2019.7, published online 05 February 2019

After publication it was noticed that the sequence of the primer 16S_BV3r was given incorrectly in Table 2 as AGTGGCGGACGGGTGAGTAA. The correct sequence is CCGCGGCTGCTGGCAC and this has been amended in the new version of Table 2.

